# Clinical predictive factors of histological chorioamnionitis: case-control study

**DOI:** 10.1016/j.heliyon.2020.e05698

**Published:** 2020-12-16

**Authors:** H. Zaidi, N. Lamalmi, L. Lahlou, M. Slaoui, A. Barkat, S. Alamrani, Z. Alhamany

**Affiliations:** a Mohammed V University, Faculty of Medicine and Pharmacy, 10170, Rabat, Morocco; b Laboratory of Pathological Anatomy and Cytology, Children's Hospital, CHU Ibn Sina, 10170, Rabat, Morocco; c Laboratory of Biostatistics and Clinical Research, Faculty of Medicine and Pharmacy, 10170, Rabat, Morocco; d Neonatology Department P5, Children's Hospital, CHU Ibn Sina, 10170, Rabat, Morocco; e Maternity Souissi, CHU Ibn Sina, 10170, Rabat, Morocco

**Keywords:** Pathology, Women's health, Obstetrics & gynecology, Pregnancy, Placenta, Anatomical examination, Intrauterine infection, Clinical chorioamnionitis, Histological chorioamnionitis

## Abstract

**Introduction:**

Histological chorioamnionitis or "intrauterine inflammation or infection" (Triple I) it is an acute inflammation of amniotic membrane, chorionic plate and umbilical cord.

**Subject:**

To assess in the event of the clinical predictive factors associated to histological chorioamnionitis.

**Methods:**

Prospective examination of 50 placentas from aberrant pregnancies, and 50 placentas from 'normal' deliveries. The Placentas analyzed by the conventional histopathology method, and the severity of chorioamnionitis was classified histologically according to the intensity and the topography of placental inflammation.

The clinical and histopathological features of the study groups were introduced into the SPSS 13 database (License University Mohammed V-Rabat).

**Results:**

36/50 placentas of aberrant pregnancies showed a histological chorioamnionitis often associated to a funisitis, and 11/50 normal placentas have shown some lesions of histological chorioamnionitis mainly grade one without funisitis.

On the other hand we noted a statistically significant association between histological chorioamnionitis and premature rupture of the membranes (PROM) over than 12h (p < 0.001).

**Conclusions:**

Our study confirmed the predominance of histological chorioamnionitis lesions in clinically suspected cases of chorioamnionitis with 72% versus 22% in the controls group.

Among the clinical parameters studied, only the premature rupture of the Membranes was shown a statistically significant association with the appearance of histological signs of chorioamnionitis.

In conclusion, chorioamnionitis is sometimes clinically silent. Morphological placental study could be a confirmation of this pathology, which is predominantly associated to PROM over than 12 h.

## 1 Introduction

The definition of histological chorioamnionitis or "inflammation or intrauterine infection" (Triple I) is acute inflammation of amniotic membranes, chorionic plate and umbilical cord most often of bacterial origin [1, 2] and it is the most common pathology during pregnancy. It is a frequent lesion, in the case of premature rupture of membranes (75%) and in the case of preterm labor with intact membranes (30%) [3]. The diagnosis of chorioamnionitis is pathological but may be suspected according to clinical data: premature rupture of membranes occurred 12 h before delivery, maternal fever over 38 °C, uterine contractions, stained amniotic fluid, fetal tachycardia greater than 180 per minute. Biological data may also suggest the diagnosis: polynucleosis greater than 20,000 per mm3, C-reactive protein (CRP) greater than 20 mg/l, and positive bacteriological examination on vaginal swab or amniotic fluid [2].

This inflammatory process is often clinically asymptomatic. It is described in only 0.9–10.5% of pregnancies before the presentation of the signs mentioned above [4].

Histologically, chorioamnionitis is characterized by the presence of neutrophils polynuclear cells of maternal and/or fetal origin in the amniotic membranes, the chorionic plate, and the umbilical cord [3, 5, 6].

This disease can have severe consequences on the mother and the newborn prematured or born at term: periventricular leukomalacia, neonatal encephalopathy, cerebral palsy, intraventricular hemorrhage, chronic lung disease, necrotizing enterocolitis [2, 7].

The purpose of this study is to investigate histological chorioamnionitis and its clinical predictors through a case-control study for the first time in Moroccan patients.

## 2 Methods

This is a case-control study, with prospective data collection, examining 100 placentas collected within the Souissi maternity Hospital of Ibn Sina Hospital of Rabat, including 50 placentas from pathological pregnancies, and 50 placentas from normal deliveries. Demographic and clinical data (maternal and neonatal), were recorded as and when patients were admitted, were collected on SPSS 13.0 database. Confidentiality of the data was ensured by assigning an anonymous number to each case.

Patients included in the study were all admitted to the Souissi Maternity Hospital of Rabat between February 2014 and October 2016. The inclusion criteria were suspicion of maternal or neonatal infection with the presence of one or more of the following signs: i)Prematurity (<37 WA), ii) Premature rupture of membranes (PROM) exceeding 12 h, maternal fever above 38 °C and iii) meconium stained amniotic fluid. The exclusion criteria included twin pregnancies and abnormal placentation (placenta accreta, percreta and prævia). In the control group, we included pregnant women with normal pregnancy and delivery at term without any complication or sign of infection.

### 2.1 Judgment criteria

#### 2.1.1 Clinical diagnosis

Histological chorioamnionitis was clinically diagnosed by the presence of fever (>38 °C) associated with one or more of the following clinical signs: premature rupture of membranes (PROM), fetal tachycardia (>180 beats per min) maternal tachycardia (>100 beats per min), uterine contractions, stained or smelly amniotic fluid.

#### 2.1.2 Histological diagnosis

Histological chorioamnionitis was diagnosed by the presence of an unaltered neutrophil infiltration with varying intensity at the amniotic membrane and placenta (maternal inflammatory response), and at the umbilical cord « funisitis» (fetal inflammatory response).

#### 2.1.3 Classification of histological chorioamnionitis

To assess the intensity of histological chorioamnionitis, we used the classification system recommended by **the Amniotic Fluid Infection Nosology Committee** and reported by *Redline et al.* in 2003 [8].

Therefore, acute inflammatory lesions of the placenta were classified into two categories: "maternal inflammatory response" and "inflammatory response fetal".The term "stage" refers to the progression of the disease based on anatomical regions infiltrated by neutrophils, and the term "grade" represents the intensity of this infiltrate. In the context of a maternal inflammatory response, stage 1 lesion is characterized by the presence of neutrophils in the chorion or subchorionic space; stage 2 refers to neutrophilic infiltration of chorionic connective tissue and/or amnion, or chorionic plaque; and stage 3 concerns necrotizing chorioamnionitis with altered neutrophil.

Grade 1 (mild to moderate) refers to small clusters of maternal neutrophils diffusely infiltrating amnion, chorionic plate, sub-chorionic fibrinoid necrosis or amnion. Grade 2 (severe) consists of three or more chorionic microabscesses defined as the confluence of neutrophils measuring at least 10 × 20 cells. Microabscesses are generally located between the chorion and the decidua, and/or under the chorionic plate. Grade 2 also refers to the presence of a continuous band of confluent neutrophils in the chorion more than 10 cells in width, occupying more than half of the subchorionic fibrinoid necrosis.

Staging and grading are also applicable to the fetal inflammation response. Stage 1 is defined by the presence of neutrophils in chorionic vessels (chorionic vasculitis) and/or the umbilical vein (umbilical phlebitis). Stage 2 is reached when neutrophils enter the wall of the umbilical artery (umbilical arteritis), with extension into Wharton's jelly. Stage 3 is characterized by the organization of neutrophils in Walton's jelly in concentric bands surrounding one or more umbilical vessels (concentric umbilical perivascular or necrotizing funisitis).

Staging is more important for assessing the severity of the inflammatory process. For example, amnion inflammation (amnionitis) is often associated with more intense fetal and intra-amniotic inflammation than the inflammation of the chorion alone.

### 2.2 Pathological examination of the placenta

Macroscopic and histological examination was carried out at the Laboratory of Anatomy and Pathological Cytology of the Children's Hospital complex Souissi CHU Ibn Sina of Rabat.Macroscopic examination was performed on the fresh tissue and then fixed according to the technical steps conventionally described by *Coulomb and col., 2005* [2].

The placentas were then fixed in 10% formalin for 24–48 h.For microscopic examination, at least three samples were taken from each placenta: cord at the proximal and distal sides, free membranes in roll, and placenta diameter from the chorale plate to the basal plate. Eventually, conventional histology was carried out.

### 2.3 Statistical analysis

Clinical and histopathological demographic characteristics of both groups were evaluated using the statistical software SPSS version 13.0 (License of the University Mohammed V-Rabat). The statistical significance was fixed at *p* < 0.05.

Descriptive and analytical analysis were performed (univariate and multivariate). For the descriptive study, the quantitative Gaussian distribution variables were expressed as mean and standard deviation and then compared by Student's t-test. Quantitative non-Gaussian distribution variables were expressed in median and inter-quartile ranges and compared by the Mann-Whitney test.

The qualitative variables were expressed in numbers and percentages then compared by exact Chi-square and Fisher tests according to the conditions of application of each.The search for clinical predictors of histological chorioamnionitis occurrence was performed by logistic regression in uni and multivariate analyzes, and variables with significant *p* were introduced in a multivariate model to study the association with histological chorioamnionitis.

### 2.4 Ethics approval and consent to participate

Our study have performed in accordance with the Declaration of Helsinki.

Respect of confidentiality and personal information of the participant was the priority during data collection and analyses, by assigning an anonymous number to each case, so no personal data was visible.

Since all analyses were made with anonymized data thus ethical approval and written informed consent was not required because of the anonymous nature of the data.

## 3 Results

### 3.1 Demographic and clinical data

In the present study, 100 placentas were collected 50 placentas with clinical signs of infection, and 50 placentas from normal delivery.

The mean age of the patients was 27.79years ±6.19, gestational age was 38.9 ± 2.4 weeks, and caesarean section was performed in 27% of our population. A history of abortion, in utero fetal death, stillbirth and other medical history was observed in only 8 patients. Clinical signs of infection such as PROM, fever and stained amniotic fluid were found in 43%, 21.6% and 17.8% of our population, respectively.

### 3.2 Frequency and severity of histological chorioamnionitis

**Figure 1:**
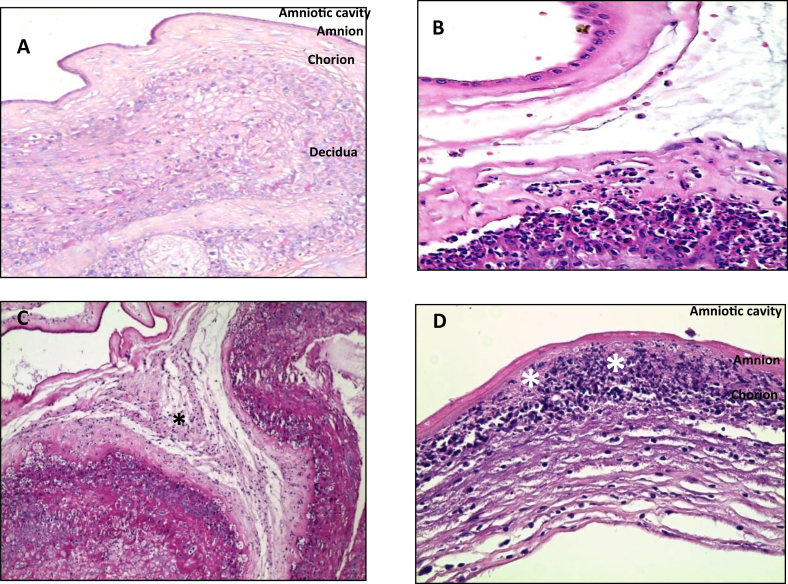
Staging of acute chorioamnionitis: Maternal inflammatory response (HE) A. Normal chorioamniotic membranes shows the absence of neutrophils; B. Acute chorionitis is stage 1 acute inflammation of the chorioamniotic membranes, in which neutrophilic infiltration is limited in the chorionic and sub-chorionic space (S1G1); C, Acute chorioamnionitis is stage 2 acute inflammation of the chorioamniotic membranes; neutrophilic migration into the amniotic connective tissue is shown (asterisk) (S2G2); D. Necrotizing chorioamnionitis is stage 3 acute inflammation of the chorioamniotic membranes, whose characteristic is the amnion epithelial necrosis (asterisk) (S3G3). ∗ Redline RW and col classification.

**Figure 2:**
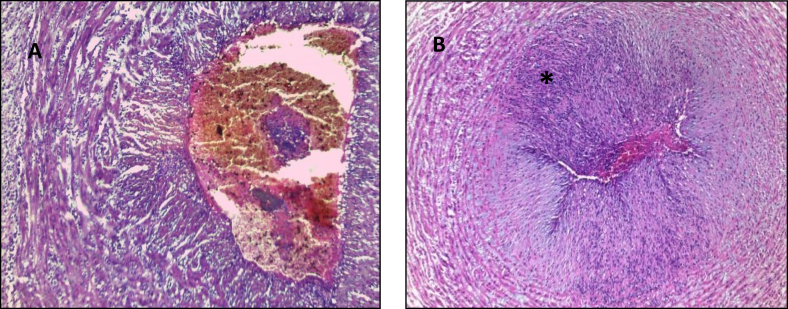
Staging of acute funisitis: Fetal Inflammatory Response (HE) A. Umbilical phlebitis shows amniotropic migration of fetal neutrophils into the muscle layer of the umbilical vein. Umbilical phlebitis represents stage 1 fetal inflammation; B. Umbilical arteritis is a stage 2 fetal inflammatory response. ∗ Redline RW and col classification.

In cases with one or more clinical signs of infection, 36/50 (72%) placentas showed histological chorioamnionitis mainly stage 2 and 3: Stage 1 (N = 9) stage 2 (N = 15) and stage 3 (N = 12), Figure 1B, C and D illustrate the main morphological lesions. Associated funisitis (N = 16) and very severe funisitis with necrosis (N = 3) (Figure 2A, B).

In the control group, 11/50 (22%) placentas showed predominantly stage 1 histological chorioamnionitis without funisitis: stage 1 (N = 9), stage 2 (N = 2) and stage 3 (N = 0) funisitis (N = 0). Figure 1A, B illustrate the main morphological lesions.

### 3.3 Comparative statistics

Histological chorioamnionitis was observed in 72% of clinically suspected cases, and only 22% in the control group. Results are shown in Table 1.

**Table 1:** Comparative table case/control.

Variable	Case (N = 50)	Control (N = 50)	p value
Maternal age (years)	28.35 ± 6.79∗	27.38 ± 5.73∗	0.47
Gestity	2.06 ± 1.56	1.80 ± 1.14	0.38
Parity	1.86 ± 1.41	1.72 ± 1.01	0.59
Temperature (°C)	37.83 ± 0.99	36.95 ± 0.19	<0.001∗∗
Gestational age (weeks)	38.08 ± 3.104	39.54 ± 1.541	0.005∗∗
Mode of delivery:			
• Vaginal	25 (50%)¥	48 (96%)	<0.001∗∗
• Cesarean	25 (50%)	2 (4%)	
Maternal medical and surgical history:			
• Yes	5 (12.9%)	3 (6%)	0.059
• No	34 (87.2%)	47 (94%)	
Premature rupture of membranes:			
• Yes	43 (86%)	0 (0%)	<0.001∗∗
• No	7 (14%)	50 (100%)	
Amniotic fluid:			
• Stained	16 (40%)	0 (0%)	<0.001∗∗
• Normal	24 (60%)	50 (100%)	
Birth weight (grams)	3157.06 ± 560.71	3250 ± 513.63	0.44
Anomaly of the newborn:			
• Yes	3 (7.7%)	0 (0%)	0.080
• No	36 (92.3%)	50 (100%)	
Apgar score:			
• 10/10	29 (85.3%)	50 (100%)	0.009∗∗
• < 10	5 (14.6%)	0 (0%)	
Histological Chorioamnionitis:			
• Yes	36 (72%)	11 (22%)	<0.001∗∗
• No	14 (28%)	39 (78%)	

Population studied in Table 1 are Moroccan population “North African Arabs”.

∗ Means ± SD.

¥ Numbers (percentage).

∗∗ significant, (p < 0.001).

A comparative statistics between the two study groups is summarised in Table 1.

### 3.4 Clinical predictors of histological chorioamnionitis

According to univariate analysis, this study showed that histological chorioamnionitis was associated with fever greater than or equal to 38 °C (*p* = 0.002), premature rupture of membranes 12 h prior to delivery (*p* = 0.001), a stained amniotic fluid (*p* = 0.003), and the mode of delivery with a (*p* = 0.002).

On the other hand, there was no statistically significant difference for gestational age, gestity and parity, nor for birth weight, with a p > 0.05 (Table 2).

**Table 2:** Univariate analysis.

Variable	OR	95% CI	p value
Maternal age (years)	1.018	[0.95–1.09]	0.61
Mode of delivery	4.87	[1.82–13.01]	0.002∗
Gestity	1.09	[0.79–1.51]	0.58
Parity	1.07	[0.74–1.53]	0.72
Maternal medical and surgical history	0.85	[0.53–1.35]	0.48
Gestational age (weeks)	2.22	[0.64–7.66]	0.2
Premature rupture of membranes	6.62	[2.74–15.99]	0.001∗
Stained amniotic fluid	7.54	[1.97–28.85]	0.003∗
Fever	2.54	[1.39–4.61]	0.002∗
Blood pressure	1.05	[0.66–1.67]	0.8
Birth weight (grams)	1	[0.99–1.00]	0.8
Apgar score	0.9	[0.70–1.24]	0.6

OR: Odds Ratio; CI: confidence interval.

∗ significant, (p < 0.001).

By adjusting variables, principally the mode of delivery, PROM, stained amniotic fluid and fever, only PROM has shown a statistically significant association with histological chorioamnionitis (*p* = 0.007). Results are shown in Table 3.

**Table 3:** Multivariate analysis.

Variable	OR	95% CI	p value
mode of delivery	1.46	[0.25–8.51]	0.6
Premature rupture of membranes	6.38	[1.66–24.38]	0.007∗
Stained amniotic fluid	1.87	[0.28–12.38]	0.5
Fever	0.89	[0.34–2.33]	0.8

OR: Odds Ratio; CI: confidence interval.

∗ significant, (p < 0.001).

## 4 Discussion

The purpose of the present study was to determine the clinical predictors of histological chorioamnionitis among Moroccan patients through a case-control study.

The univariate analysis showed the predominance of histological chorioamnionitis lesions in C-section delivery, PROM 12 h prior to delivery, stained amniotic fluid and fever greater than or equal to 38 °C. Thus in the multivariate analysis only the PROM shows a statistically significant association with a histological chorioamnionitis.

Several studies have shown that the incidence of histological chorioamnionitis is much higher if the infection is clinically diagnosed [9, 10]. PROM is associated with histological chorioamnionitis in 60% of cases [11], and in several studies, it is considered as a clinical predictor of this condition [8, 12].

In the study conducted by *Elimian and col.,* 61% of women with PROM had histological signs of chorioamnionitis [13]. For *Rebecca and col.* PROM is strongly associated with the appearance of histological signs of chorioamnionitis (p < 0.001) [14].

In 2013, *Nasef and col.* noted the association of PROM with histological chorioamnionitis (47% vs 17%) [15]. Accordingly, in the series of *Hui-Chung et al.* and *Park et al.* authors have found (51.85% vs 21.62%) and (62% vs 39%), all these results were statistically significant (p < 0.05) [16,17].

In multivariate analysis, several authors did not find any statistically significant differences concerning the following variables: stained amniotic fluid, fever, and the mode of delivery even if these parameters were associated with histological chorioamnionitis in univariate analysis [8, 14, 15].

Regarding the influence of other clinical parameters on the risk of developing histological chorioamnionitis, such as gestity and parity, blood pressure and maternal medical and surgical history, they didn't show any statistically significant difference even when potential confounding factors were controlled by multivariate logistic regression [13, 18].

In this study, prematurity did not show a statistically significant difference. However, histological chorioamnionitis is observed in 2–4% of deliveries at term, and in 40–70% of premature deliveries (before 37 weeks) [12, 19]. Our result is due to the low rate of premature babies in our series (16%).

The Apgar score and neonatal weight did not show a statistically significant difference between cases with histological chorioamnionitis and controls (p > 0.05) [7,12,13].

The presence of inflammatory infiltrate in amniotic membranes and placenta might be due to bacterial chemotactic factors and the concentration of chemokines and complement components that activate and promote the migration, adhesion and transmigration of circulating maternal neutrophils in the intervillous space in the chorion or sub-chorionic fibrin [8]. Consequently, the first reliable histological indicator of a maternal inflammatory response to infection is a diffuse neutrophil band either at the chorion or at the subchorionic space (stage 1). Later, neutrophils cross the chorion to enter the chorionic connective tissue and/or amnion, resulting in acute histologic chorioamnionitis (stage 2).

The normal life span of a tissue neutrophil is 24–36 h; although this period may be modulated by some survival factors being recently characterized [20, 21]. Apoptosis and neutrophil fragmentation are among the defining features of stage 3 of the maternal inflammatory response, called necrotizing chorioamnionitis.

In our study, stages 2 and 3 of maternal inflammatory response were most frequently found in the placentas of patients with clinical signs of infection (especially PROM).

The fetal inflammatory response to histological chorioamnionitis is defined by the presence of neutrophils in the muscle walls of the veins or arteries of the chorionic plate (chorionic vasculitis) and/or the umbilical cord (Funisitis). The prevalence of fetal inflammatory response in histological chorioamnionitis increases with the presence of clinical signs of infection (especially PROM) (p < 0.06), and with advanced stages and severity of maternal inflammatory response (stage 2 and 3) [22].

In histological chorioamnionitis, the origin of neutrophils found in maternal membranes and chorionic vessels (fetal) was investigated by fluorescence in situ hybridization (FISH) analysis of the Y chromosome [23]. This technique showed that most of the amniotic fluid neutrophils and fetal compartments in contact with the amniotic fluid were of fetal origin [24, 25].

Histological chorioamnionitis does not always seem to be related to infection, it may be "sterile" inflammation due to extra placental causes. Several studies confirm this hypothesis. Some clinicians also currently use biological predictive and prognostic factors. The best would be the evaluation of MMP-8 and IL6 levels [26].Thus, according to our results and with the increasing rate of maternal-fetal infection, the histological examination of the placenta represents a non-invasive, simple and indispensable procedure to confirm the diagnosis of not only clinically suspected cases, but also infra-clinically cases [27].

## 5 Conclusion

Histological Chorioamnionitis is sometimes clinically silent which can be easily confirmed by anatomo-pathological analysis of the placenta.

According to our study and the literature review, clinical predictive markers for confirmed histological chorioamnionitis is premature rupture of the membranes 12 h prior to delivery, with or without any associated clinical signs. The presence of inflammation is more reproducible than the exact determination of stage and grade.

The clinical implications of histological chorioamnionitis in childbirth and maternal and neonatal morbidity and mortality should be further investigated to measure it short-term and long-term consequences. Larger multi-centric studies are needed to better understand histological chorioamnionitis.

## Declarations

### Author contribution statement

Z. Alhamany: Conceived and designed the experiments; Analyzed and interpreted the data.

H. Zaidi: Conceived and designed the experiments; Performed the experiments; Analyzed and interpreted the data; Wrote the paper.

N Lamalmi: Analyzed and interpreted the data.

A. Barkat, S. Amrani, M. Slaoui and L. Lahlou: Contributed reagents, materials, analysis tools or data.

### Funding statement

This research did not receive any specific grant from funding agencies in the public, commercial, or not-for-profit sectors.

### Data availability statement

Data will be made available on request.

### Declaration of interests statement

The authors declare no conflict of interest.

### Additional information

No additional information is available for this paper.
